# An innovative treatment for lung cancer using gene-engineered human-induced pluripotent stem cell-derived natural killer cells

**DOI:** 10.1007/s00262-026-04370-7

**Published:** 2026-03-31

**Authors:** Yuka Sato, Kumiko Goto, Shigehiro Yagishita, Kotoko Miyata, Noriko Uesugi, Yu-suke Torisawa, Yoichi Naritomi, Ryuta Takahashi, Rumiko Sho, Yuriko Takeno, Kenji Kurachi, Masashi Yamada, Yasuyuki Higashi, Hironobu Kimura, Akinobu Hamada, Fusako Nishigaki, Kouichi Tamura

**Affiliations:** 1 Kobe Research Institute, HEALIOS K.K, Kobe, Japan; 2 Division of Molecular Pharmacology, National Cancer Center Research Institute, Tokyo, Japan

**Keywords:** iPSC (induced pluripotent stem cell), NK (natural killer) cells, Genetic engineering, Cell therapy, Lung cancer, PDX (Patient-derived xenograft)

## Abstract

Various therapeutic approaches have been developed for lung cancer, including chemotherapy, radiation therapy, and immune checkpoint inhibitors. However, these approaches, including chimeric antigen receptor (CAR)-T cell therapy, have shown limited efficacy against solid tumors, especially in advanced disease.

To enhance the therapeutic effect, we focused on the multiple effects of a new modality of cell therapy and created engineered natural killer (eNK) cells, which are gene-engineered induced pluripotent stem cell (iPSC)-derived NK cells armed with CC motif ligand 19 (CCL19), CC chemokine receptor type 2B (CCR2B), high-affinity cluster of differentiation 16 (CD16), interleukin (IL)-15, and natural killer group 2, member D (NKG2D)-DNAX-activating protein 10 (DAP10) complex. In vitro studies showed that eNK cells exhibit significant long-lasting cytotoxicity and antibody-dependent cell-mediated cytotoxicity (ADCC) against human lung cancer cell lines. In vivo, eNK cells achieved near-complete tumor regression in orthotopic and subcutaneous cell line-derived xenograft (CDX) models. In contrast, in patient-derived xenograft (PDX) models, eNK cells demonstrated modest tumor growth inhibition (28% reduction) as monotherapy and significantly enhanced efficacy (53% inhibition) in combination with cetuximab via antibody-dependent cellular cytotoxicity. In treated PDX tumors, human CD45-positive cells were detected within the tumor parenchyma, supporting intratumoral presence of administered human cells.

These findings support the potential contribution of the five-gene modifications in enhancing tumor homing, persistence, and cytotoxicity in solid tumor treatment. This study underscores the potential of eNK cells as a novel, “off-the-shelf” allogeneic therapy for refractory solid tumors, including lung cancer.

**Supplementary Information:**

The online version contains supplementary material available at 10.1007/s00262-026-04370-7.

## Introduction

There has been remarkable progress in the research and development of innovative therapeutic methods such as immune checkpoint inhibitors, cell therapy, gene therapy, and nucleic acid medicine therapy. However, none of these therapies address the challenges of treating refractory solid cancers, and more promising therapeutic methods are needed.

Regarding innovative cell therapies, various chimeric antigen receptor (CAR)-T cell therapies have been investigated in non-clinical and clinical trials targeting solid tumors [1, 2]; however, no breakthrough effects have been achieved and their efficacy against solid tumors is still limited. On the other hand, natural killer (NK) cells are attracting attention because they attack targets in a non-HLA-restricted manner, have the advantage of not causing graft-versus-host disease (GVHD), and are safer than T cells [3]. Our concept is to create groundbreaking cell therapies that could broadly recognize cancer cells and kill them directly, rather than targeting limited numbers of targets with CAR-T cells for highly heterogeneous solid tumors. Engineered natural killer (eNK) cells have the potential to be highly effective in treating various refractory solid cancers.

**Fig. 1:**
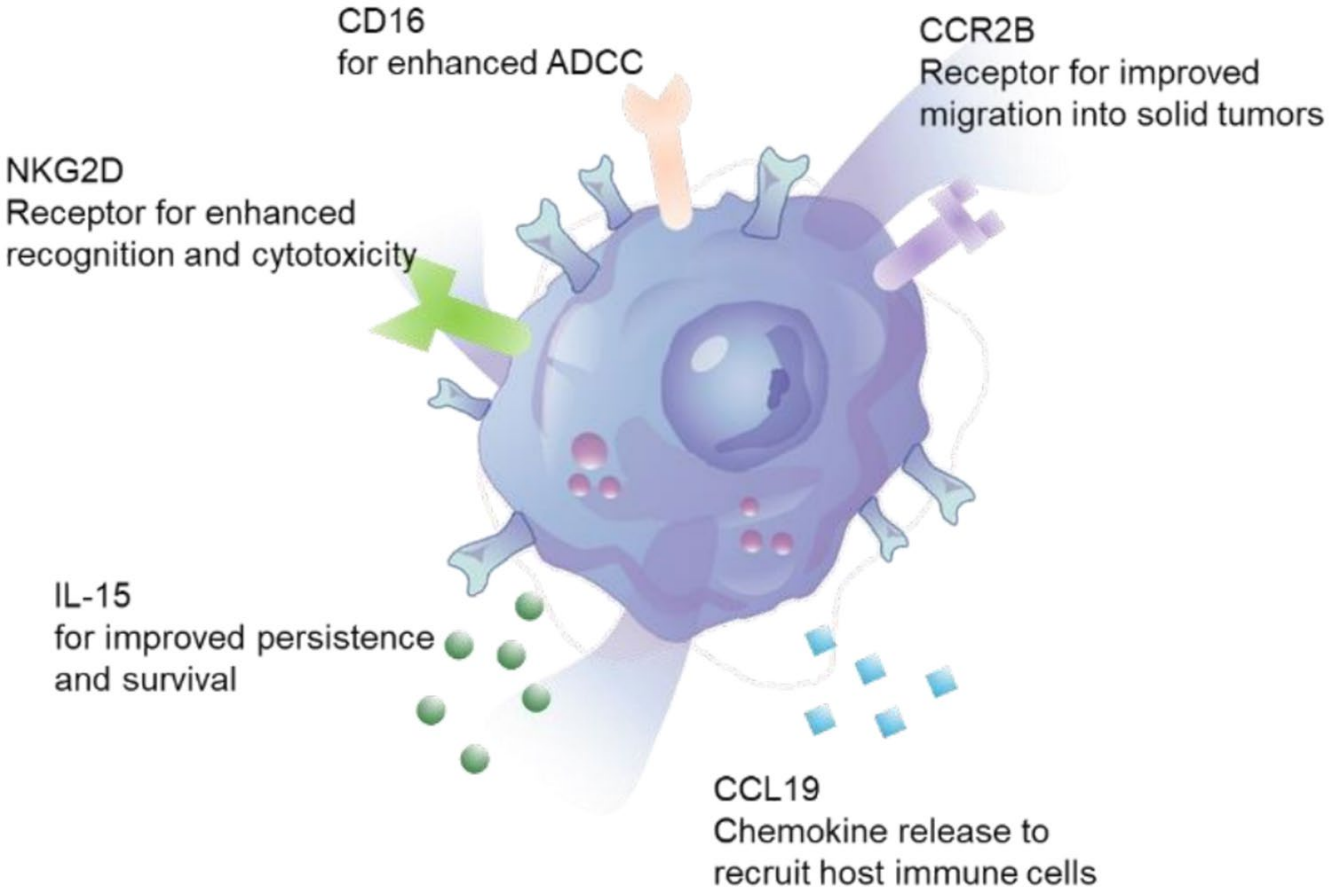
Visual representation of eNK cellular structures and processes

The interaction of cancer cells with immune cells in the tumor microenvironment (TME) is a crucial factor in cancer immunotherapy. To enhance the homing and infiltration of immune cells into the TME, we transfected CC motif ligand 19 (CCL19) [4, 5] to recruit patient immune cells to tumor tissue, and CC chemokine receptor type 2B (CCR2B) [6, 7] to enable eNK cells to migrate toward CCL2-expressing cancer cells. For immunological cells to be effective, they need to have strong cytotoxic activity against cancer cells and persist in the tumor and surrounding tissues [8]. To achieve suitable lymphocyte persistence and activation levels [9], we transfected interleukin (*IL*)*15* gene, as well as natural killer group 2, member D (*NKG2D*), and DNAX-activating protein 10 (*DAP10*) genes to improve cancer cell detectability. Moreover, in the case of NK cells, antibody-dependent cellular cytotoxicity (ADCC) activity can be effectively utilized, so we attempted to enhance the expression of cluster of differentiation (CD)16 and increase the value of our therapy by combining it with antibodies [10]. As a result of these creative hypotheses, we developed eNK cells, which are gene-engineered human-induced pluripotent stem cell (hiPSC)-derived NK cells armed with NKG2D, IL-15, CD16, CCL19, and CCR2B molecules (Fig. 1) [11].

Our previous study showed that these genetic modifications enhanced tumor homing, immune cell recruitment, and in vivo persistence. Furthermore, eNK cells exhibited significantly superior antitumor effects compared to unmodified iPSC-derived NK cells (iNK) in both in vitro and in vivo studies, including studies utilizing the H1975 orthotopic lung cancer model [11].

Building on these findings, in this study, we further explore the therapeutic potential of our eNK cells, focusing on lung cancer as a model system. Lung cancer is the leading cause of cancer-related deaths worldwide and consistently ranks high when it comes to annual mortality rates for both men and women. Lung cancer is often diagnosed when it is in an advanced stage. Although the number of treatment options, including chemotherapy drugs, immune checkpoint inhibitors, and antibodies, is increasing, almost no effective therapy options are available for patients with advanced disease. On the other hand, it has been reported that intratumoral injection into solid tumors is a promising strategy for enhancing the efficacy of cancer treatment and avoiding systemic adverse effects [12, 13]. First, because there have been few successful cases of systemic administration of cell therapy, we investigated whether eNK cells can exert an anti-solid tumor effect by injecting them directly into the tumor mass. We evaluated this strategy using cell line-derived xenografts (CDX) and patient-derived xenografts (PDX) and obtained results that are expected to be highly accurate for clinical application. We hypothesized that eNK cells would exhibit similar organ-dependent distribution characteristics regardless of whether they are administered intravenously or directly into the tumor mass. This hypothesis is based on the expectation that the therapeutic efficacy observed in CDX and PDX models could also be achieved through systemic administration, thereby underscoring the clinical relevance of eNK cell therapy in real-world applications. As hypothesized, eNK cells showed robust antitumor activity in lung cancer models. The in vivo persistence and antitumor effects of eNK cells can be enhanced by gene transfer and further enhanced by combining gene transfer with antibodies that exert ADCC mediated by transfected CD16 cells.

## Materials and methods

### Cell lines and cell culture

The lung cancer cell lines used were A549, NCI-H1975-Luc (luciferase expressing NCI-H1975), NCI-H460-Luc (luciferase expressing NCI-H460), Lu99, NCI-H520, and SBC-3. A549-GFP and NCI-H1975-Luc-GFP (H1975-GFP) cells were established by transfection of EF1α-GFP-IRES-Puro or Hygro-hGHpA vector using the piggyBac transposon system into each cell type and subsequent cloning. A549 and NCI-H520 cells were purchased from American Type Culture Collection. NCI-H1975-Luc, NCI-H460-Luc, Lu99, and SBC-3 cells were purchased from National Institute of Biomedical Innovation, Health, and Nutrition (Osaka, Japan). These cells were grown in monolayer culture in Ham’s F-12 K (Kaighn’s) medium (A549 and A549-GFP), RPMI 1640 (NCI-H1975-Luc, NCI-H1975-GFP, NCI-H460-Luc, Lu99, NCI-H520), or MEM (SBC-3) supplemented with 10% fetal bovine serum (FBS) (Biosera, Cholet, France), in a humidified incubator set to 37 °C and gassed with 5% CO_2_.

J-PDX_E0050 tumor was provided by the National Cancer Center J-PDX Library, Japan (Tokyo, Japan), and maintained by subcutaneous transplantation in NOD/Shi-scid IL-2R gamma (null) (NOG) mice.

### Animals

NOG mice [14] were purchased from In-Vivo Science Inc. (Kawasaki, Japan) and maintained under specific pathogen-free conditions with ad libitum access to water and food.

CDX model experiments were performed in accordance with the relevant institutional and national guidelines and regulations and were approved by the Institutional Animal Care and Use Committee of HEALIOS K.K.

PDX model experiments were conducted at Mediford Corporation (Tokyo, Japan) (formerly LSI Medience Corporation, Tokyo, Japan). The experiments were approved by the Ethics Committee in accordance with the “Ethics Review Regulations for Research, etc. Involving the Handling of Human Samples and Human Information”, by the Safety Committee in accordance with the “Regulations of the Genetically Modified Organisms Safety Committee (Kumamoto)”, and by the Animal Experimentation Committee and the head of the institution in accordance with the “Guidelines for Animal Experiments”. The use of human-derived PDX was carried out in accordance with the “Ethical Guidelines for Medical Research Involving Human Subjects”.

### Lactate dehydrogenase release assay

Lung cancer cells were seeded at 3.75 × 10^4^ cells/well in a 96-well plate and incubated overnight. eNK cells were co-cultured with tumor cells at effector/target (E/T) ratios ranging from 0.3 to 10 for 4 h at 37 ℃ in an atmosphere of 5% CO_2_. After co-culture with eNK cells, supernatants were collected, and lactate dehydrogenase (LDH) activity released from damaged cells was measured using an LDH cytotoxicity assay kit (Dojindo Molecular Technology, Kumamoto, Japan). Cytotoxicity (% of lysis) was calculated by using the following formula. For determining maximal LDH release, cell lysis was induced by 1% Triton X-100 solution.

$$Cytotoxicity\left( {{{\% of lysis}}} \right) = \frac{{\left( {{\text{Experimental LDH release}} - {\text{Spontaneous LDH release of effector cells}}} \right)}}{{\left( {{\text{Maximum LDH release }} - {\text{Spontaneous LDH release of target cells}}} \right)}} \times 100$$.

### Incucyte cytotoxicity assay

To generate tumor spheroids, A549 and H1975 cells stably expressing green fluorescent protein (GFP) were seeded at 1.0 × 10^4^ cells/well in a 96-well round-bottom plate (Corning, NY, USA) in Ham’s F-12 K (Kaighn’s) medium (A549-GFP) or RPMI 1640 (H1975-GFP) (Thermo Fisher Scientific, Waltham, MA, USA) supplemented with 10% FBS (Biosera). After centrifugation for 10 min at 130 × g, plates were cultured in an incubator at 37℃ and 5% CO_2_ for 4 days. Thirty thousand eNK cells were added to the wells containing one tumor spheroid in AIM-V medium (Thermo Fisher Scientific) supplemented with 5% FBS (Merck, Darmstadt, Germany), 50 ng/mL stem cell factor (SCF) (PeproTech, Cranbury, NJ, USA), and 50 ng/mL IL-15 (PeproTech) in the absence or presence of 2.5 μg/mL cetuximab (Cmab; Merck) and 3 μg/mL necitumumab (Nmab; Nippon Kayaku Co., Ltd., Tokyo, Japan). Cytotoxicity was monitored at 120-min intervals using the Incucyte SX5 imaging system (Sartorius, Göttingen, Germany).

### In vivo persistence assay

A total of 5.0 × 10^6^ eNK cells were intravenously administered to NOG mice. Peripheral blood, lung, liver, and spleen samples were collected on 1, 7, 14, and 28 days after administration. The genomic DNA (gDNA) was extracted from the peripheral blood and tissues using a DNeasy Blood & Tissue Kit (Qiagen, Hilden, Germany). The DNA content of eNK cells per gDNA content in each sample was determined by qPCR using an *Alu* sequence-specific primer set [15]. On the final day, mice were anesthetized by inhalation of vaporized isoflurane and euthanized by exsanguination from the inferior vena cava.

### In vivo antitumor activity assay

NOG mice were injected intravenously or subcutaneously with H1975-Luc or A549-Luc cells. However, any models in which cancer cells engrafted in organs other than the lungs were excluded from the study. hIL2 (PeproTech) and hIL15 were intraperitoneally injected to encourage or enhance persistence of eNK cells.

An orthotopic lung cancer model was established by intravenous injection of tumor cells. Tumor progression was assessed by bioluminescence imaging using the IVIS Spectrum system (PerkinElmer, Shelton, CT, USA). For subcutaneous tumors, the length (L), width (W), and height (H) were measured using calipers. Tumor volume (mm^3^) was calculated using the following formula: L × W × H × π/6.

For histopathological analysis, tumors were collected from tumor-bearing mice in the satellite group that did not receive eNK cells treatment. Tissue collection was performed on the first day of eNK cells administration, following euthanasia under anesthesia and exsanguination.

H&E staining and Ki67 immunostaining using anti-Ki-67 antibody (clone: MIB-1, Dako) were performed at Sapporo General Pathology Laboratory Co., Ltd. (Sapporo, Japan).

### PDX model

The experiments were conducted at Mediford Corporation in research facilities that had obtained AAALAC International Certification (Tokyo, Japan). Human lung cancer PDX lines provided by the National Cancer Center Japan (Tokyo, Japan) were subcutaneously implanted into NOG mice. eNK cells and Cmab were administered three times a week for three weeks intratumorally and intraperitoneally, respectively. hIL2 was intraperitoneally injected to encourage or enhance persistence of eNK cells. The tumor size was measured using a caliper, and the estimated tumor volume was calculated using the following formula. Tumor volume (mm^3^) = W (mm) × L (mm) × L (mm) × 1/2.

Epidermal growth factor receptor (EGFR) immunostaining using anti-EGFR antibody (clone: D38B1, Cell Signaling) was performed at Mediford Corporation (Tokyo, Japan) (formerly LSI Medience Corporation, Tokyo, Japan).

### Immunohistochemistry of PDX tumors

PDX tumors were collected on Day 35 (16 days after the last administration) and processed for histological analysis. Tumor sections were stained with hematoxylin and eosin (H&E). Immunohistochemistry was performed at the National Cancer Center Research Institute, Japan, using standard protocols on formalin-fixed, paraffin-embedded sections. Sections were stained with anti-human CD45 antibody (clone D9M81, Cell Signaling Technology, Danvers, MA, USA) and anti-human COX IV antibody (clone 3E11, Cell Signaling Technology). Immunoreactivity was visualized using a chromogenic detection method and counterstained with hematoxylin. Whole-slide images were acquired using a Hamamatsu NanoZoomer digital slide scanner (Hamamatsu Photonics, Shizuoka, Japan).

### Qualitative assessment of intratumoral CD45-positive cell clusters

Whole-slide images of human CD45 immunohistochemistry were evaluated in a blinded manner. The presence of clustered human CD45-positive cells within viable tumor parenchyma was recorded as a binary outcome (present/absent) for each tumor. “Present” was defined as at least one intratumoral cluster composed of multiple CD45-positive cells, whereas isolated single cells or background staining alone were scored as “absent.” Two independent evaluators independently scored each tumor in a blinded manner.

### Statistical analysis

All data were presented as the mean ± standard deviation (SD), unless otherwise stated. The Dunnett multiple comparison test and Student *t* test were performed as statistical significance examinations using JMP® software (SAS Institute Japan Ltd.) and EXSUS software (SAS Institute Japan Ltd.).

## Results

### The cytotoxicity of eNK cells and the contribution of the gene engineering of CD16 in vitro

**Fig. 2:**
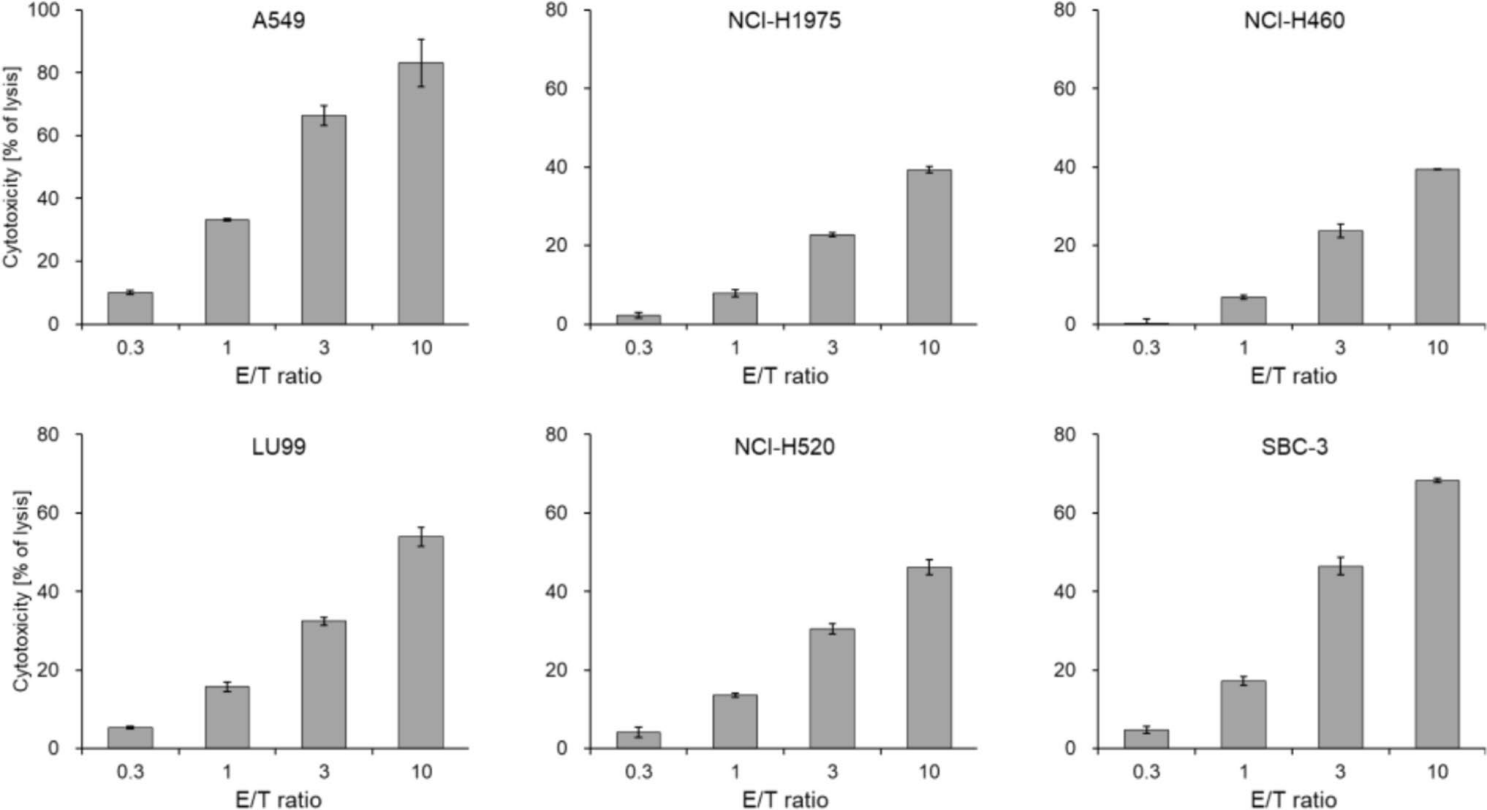
Cytotoxicity of eNK cells against human lung cancer cells. The cytotoxicity of eNK cells against human lung cancer cell lines was evaluated using the LDH release assay. eNK cells were cultured for 3 days after thawing and co-cultured with each cell line at the indicated E/T ratios. All experiments were performed in technical triplicate. Supernatants were collected after 4 h of co-culture, and LDH activity was measured. Data are shown as mean ± SD. E/T, effector to target; LDH, lactate dehydrogenase

First, using the LDH release assay, we evaluated the cytotoxicity of eNK cells against A549 (wild-type EGFR), NCI-H1975-Luc (mutant EGFR; L858R, T790M), NCI-H460-Luc (PIK3CA mutant), LU99 (wild-type EGFR), NCI-H520 (wild-type EGFR), and SBC-3 cells. eNK cells showed dose-dependent cytotoxicity in all lung cancer cell lines (Fig. 2).

**Fig. 3:**
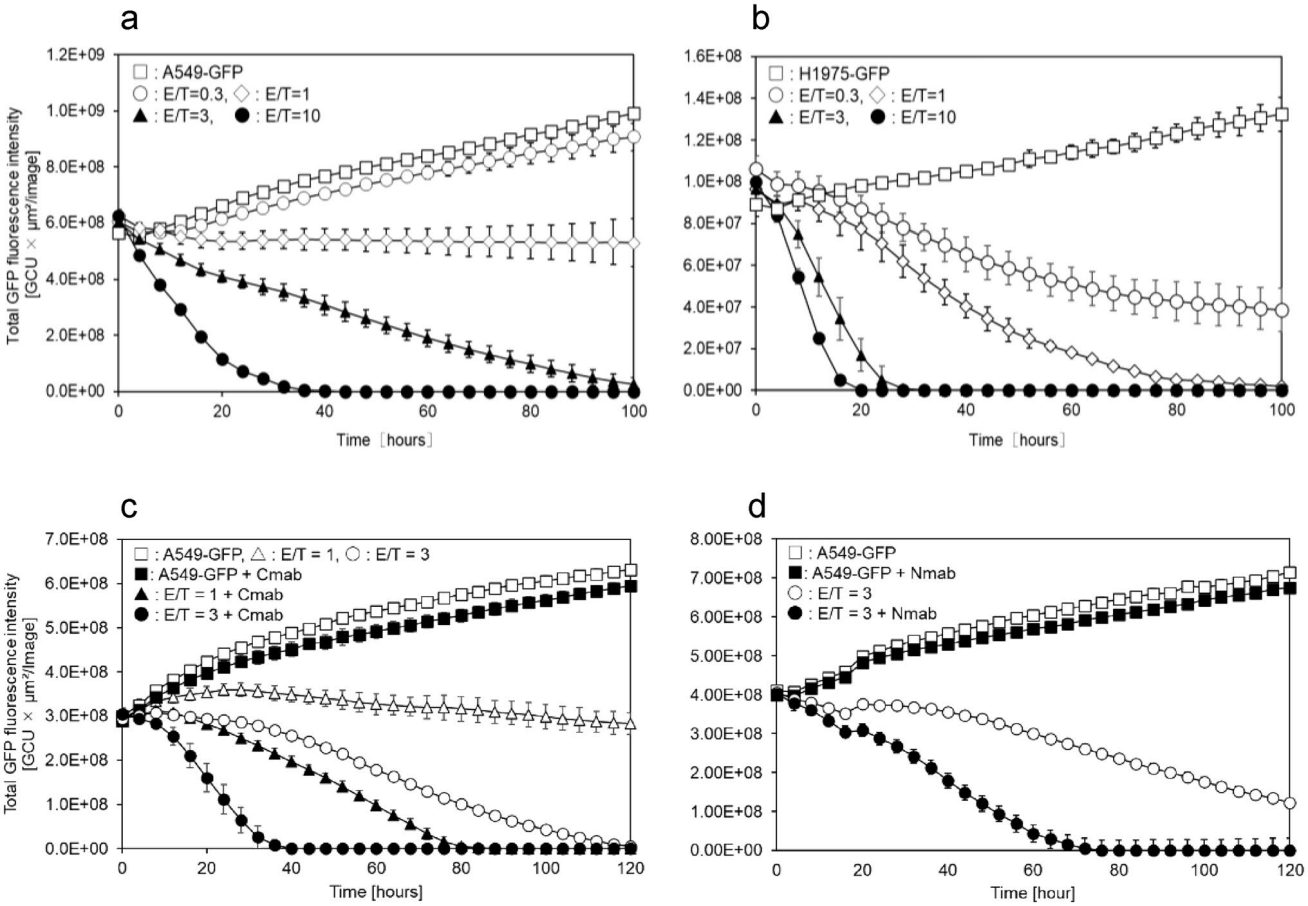
Cytotoxicity of eNK cells against A549 and H1975 lung cancer spheroids. The cytotoxicity of eNK cells against A549-GFP (**a, c, d**) and H1975-GFP (**b**) spheroids was evaluated using the Incucyte cytotoxicity assay. eNK cells were cultured for 3 days after thawing and co-cultured with each target spheroid. E/T ratios were calculated from the seeded number of A549-GFP cells or H1975-GFP cells followed by the formation of spheroids. Cmab (2.5 μg/mL) (**c**) or Nmab (2.5 μg/mL) (**d**) was added to the target spheroids prior to eNK addition. All experiments were performed in technical triplicate. Data are shown as mean ± SD. E/T, effector to target; Cmab, cetuximab; Nmab, necitumumab

Next, using the Incucyte cytotoxicity assay, we evaluated the sustained cytotoxicity of eNK cells. A549-GFP and NCI-H1975-GFP cell spheroids were, respectively, incubated with eNK cells at E/T ratios ranging from 0.3 to 10 and monitored for cell death by measuring GFP fluorescence intensity using the Incucyte live-cell imaging system. eNK cells were clearly cytotoxic for A549-GFP cells at an E/T ratio of 3 and inhibited their growth at an E/T ratio of 1. Against NCI-H1975-GFP cells, eNK cells were cytotoxic at an E/T ratio of 0.3 or more. Moreover, eNK cells retained cytotoxic activity until 120 h after the treatment (Fig. 3a, b).

We evaluated the ability of eNK cells to induce ADCC against a wild type-EGFR-positive tumor cell line (A549) via Cmab [16] and Nmab [17] binding to FcγRIII (CD16a). A549-GFP spheroids were incubated with eNK cells and Cmab or Nmab (Fig. 3c, d). Cmab and Nmab alone were not cytotoxic; however, the addition of these antibodies further enhanced the cytotoxicity of eNK cells.

### The antitumor effects of intratumoral eNK cell administration on the subcutaneously transplanted lung cancer cell line, H1975-Luc

**Fig. 4:**
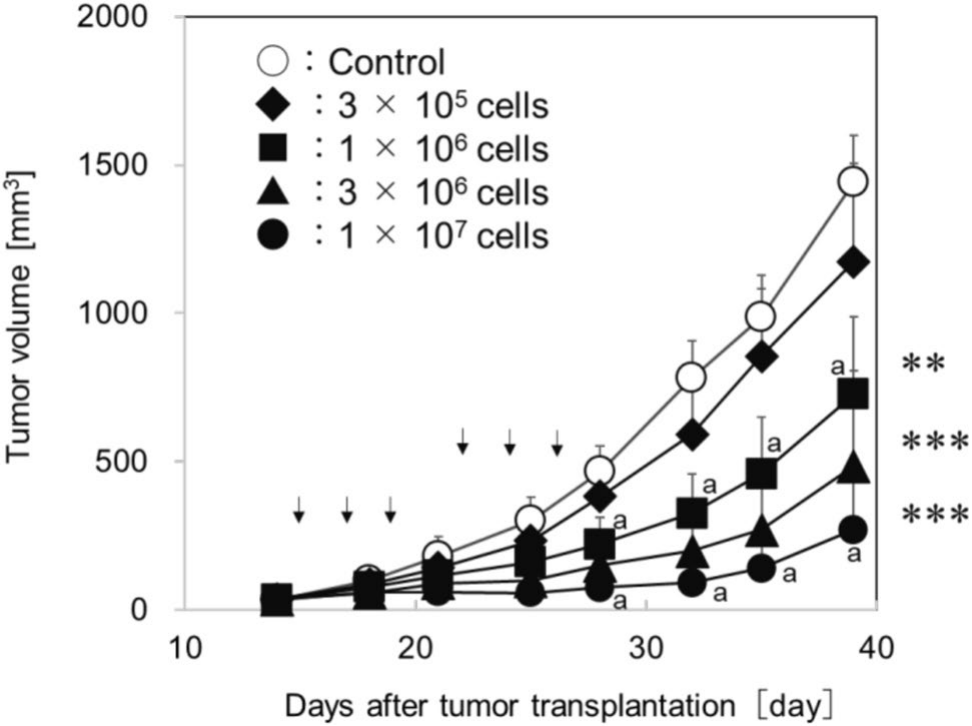
Antitumor effects of eNK cells intratumoral treatment on H1975-Luc engrafted mice. NOG mice were subcutaneously injected with 5 × 10^5^ H1975-Luc tumor cells (day 0). eNK cells at indicated dose were intratumorally administered three times a week for 2 weeks from day 14. Data are shown as mean ± SD. For all groups, *n* = 5 except at those times marked “a” (*n* = 4). Arrows depict the day of eNK cells administration. **; *p* < 0.01, ***; *p* < 0.001 vs control (vehicle) (Dunnett multiple comparison test)

We examined whether the intratumoral route of injection affects the antitumor effect of eNK cells on subcutaneously transplanted H1975-Luc, because it had been reported that intratumoral injection into solid tumors was a promising strategy for enhancing cancer treatment efficacy [12, 13]. To examine whether eNK cells could inhibit tumor growth, H1975-Luc cells were transplanted subcutaneously in NOG mice (day 0), and eNK cells were administered intratumorally three times a week for two weeks starting on day 14 after tumor transplantation. The results clearly showed that eNK cells dose dependently suppress tumor growth (Fig. 4).

### The effects of eNK cells in the PDX mouse model derived from a lung cancer patient

**Fig. 5:**
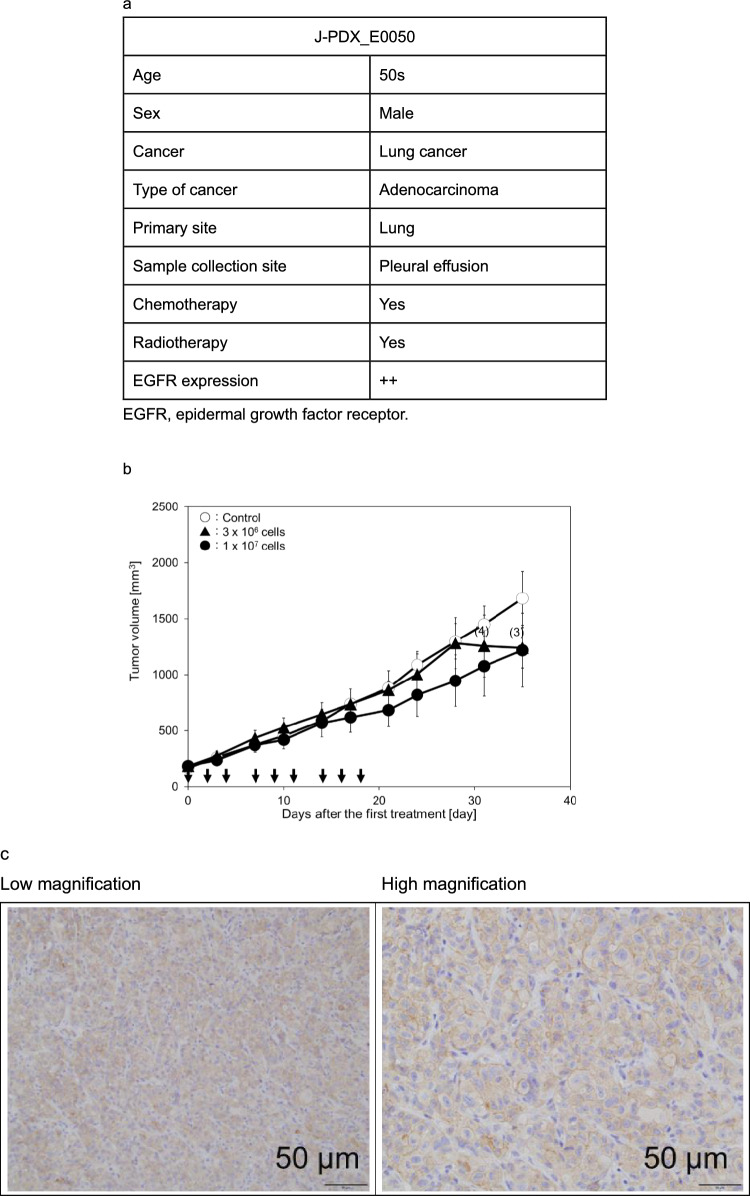

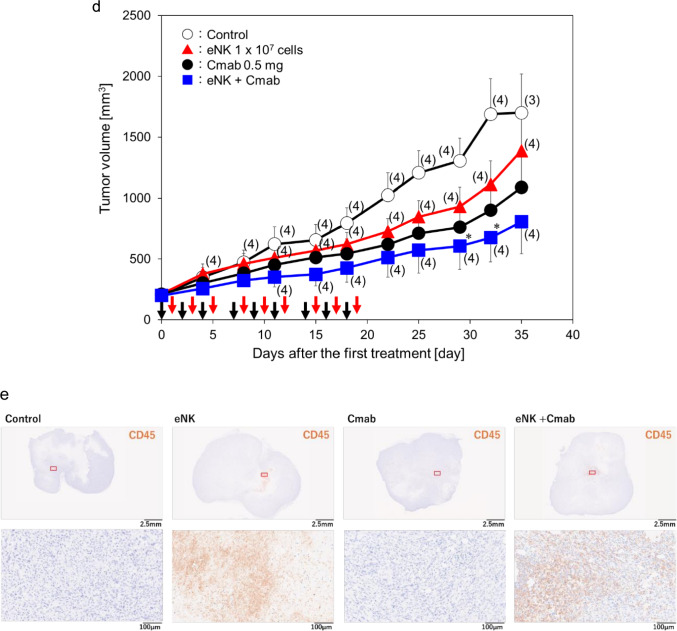
Effects of eNK cells on the PDX mouse model. **a** Characterization of J-PDX_E0050. **b** The tumor mass derived from a patient was maintained in mice, then cut into pieces (approximately 2–3 mm in size), and finally implanted subcutaneously into NOG mice. When the tumors grew to approximately 200 mm^3^, the mice were randomized (day 0). Arrows depict the day of eNK cells administration. **c** EGFR expression of tumor tissues in J-PDX_E0050-bearing NOG mice. **d** eNK cells (red arrows) and Cmab (black arrows) were administered three times a week for 3 weeks from day 0 and day 1, respectively. Data are shown as mean ± SD. For all groups, *n* = 5 except where the number of animals is indicated in parenthesis. *; *p* < 0.05 vs control (vehicle) (Student *t* test) PDX, patient-derived xenograft; NOG, NOD/Shi-scid IL-2R gamma (null); EGFR, epidermal growth factor receptor; Cmab, cetuximab. **e** Representative whole-slide images of immunohistochemistry for human CD45 in tumors harvested on Day 35 (16 days after the last administration). Sections were stained with anti-human CD45 antibody (clone D9M81, Cell Signaling Technology). Whole-slide images were acquired using a Hamamatsu NanoZoomer digital slide scanner (Hamamatsu Photonics, Shizuoka, Japan). Boxes indicate the regions shown at higher magnification. Scale bars, 2.5 mm (upper panels) and 100 µm (lower panels)

To improve the predictability of tumor growth inhibition by eNK cells in clinical applications, we examined the effects of eNK cells in a high-predictive-value PDX mouse model. The J-PDX_E0050 tumor used in the PDX mouse model (Fig. 5a, non-small cell lung cancer, adenocarcinoma) was resistant to chemotherapy and radiotherapy.

eNK cells at a dose of 1 × 10^7^ cells/mouse reduced tumor volume by 28% on day 35 in the PDX model, demonstrating modest tumor growth inhibition as monotherapy (Fig. 5b). Immunohistochemical staining was performed on the tumor tissues, which clearly expressed EGFR (Fig. 5c). Next, the combined effect of eNK cells and Cmab was examined based on ADCC. Injection of eNK cells at a dose of 1 × 10⁷ cells/mouse and Cmab at a dose of 0.5 mg/body into mice bearing PDX tumors resulted in 18% and 36% inhibition of tumor growth on day 35, respectively. The combination of eNK cells with Cmab further enhanced tumor growth inhibition to 53%, likely due to ADCC-mediated effects (Fig. 5d).

### Histological confirmation of human CD45-positive cells in treated PDX tumors

To directly assess whether administered human cells were present within PDX tumors after treatment, we performed immunohistochemistry on tumors harvested on Day 35 (16 days after the last administration) using an anti-human CD45 antibody. Human CD45-positive cells were clearly detected within the tumor parenchyma in eNK-treated tumors, showing clustered localization inside the tumor. In contrast, human CD45-positive cells were almost absent in tumors from the vehicle and cetuximab-only groups. The distribution pattern of human CD45-positive cells in the combination group was comparable to that in the eNK monotherapy group (Fig. 5e). Clustered human CD45-positive cells were observed in 4/4 tumors in the eNK group and 4/4 tumors in the combination group, whereas they were almost absent in 0/3 vehicle tumors and 0/5 cetuximab-only tumors. Because NOG mice do not possess endogenous human immune cells, the detected human CD45-positive cells are considered to represent administered eNK cells.

These results suggested that eNK cells could be effective both as a single agent and in combination with antibodies in clinical practice.

### Persistence of eNK cells in normal NOG mice

**Fig. 6:**
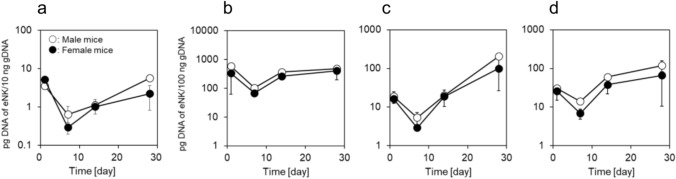
Persistence of eNK cells in NOG mice. eNK cells at a dose of 5 × 10^6^ cells were injected intravenously into male and female NOG mice (Day 0). On days 1, 7, 14, and 28, blood and organs were collected and the DNA content of eNK cells was determined by a human-specific Alu sequence qPCR assay. Panels show the concentration profile of eNK cells in blood (**a**), lungs (**b**), liver (**c**), and spleen (**d**). Lower limit of quantification (LLOQ): 0.12 pg of DNA from eNK cells/10 or 100 ng of gDNA. Data are shown as mean ± SD (*n* = 3 mice). NOG, NOD/Shi-scid IL-2R gamma (null)

eNK cells were administered intravenously to normal NOG mice, and its pharmacokinetics were investigated. eNK cells were injected intravenously at a dose of 5 × 10^6^ cells in male and female NOG mice. Human-specific Alu sequence analysis using qPCR was conducted in gDNA extracted from blood and organs collected on day 1, 7, 14, and 28 after injection. The blood concentration of human-specific Alu sequences decreased 1 to 7 days after administration and then increased on days 14 and 28 after administration (Fig. 6a). The time-dependent patterns of changes in concentration in the lungs, liver, and spleen were similar to those in the blood (Fig. 6b, c, d). In addition, eNK cells were most abundantly distributed in the lungs among the organs evaluated, suggesting that they are efficiently distributed to the sites of lung cancer involvement and exhibit antitumor effects.

### Antitumor effects of eNK cells against the human lung cancer cell line, H1975-Luc, intravenously injected into an orthotopically transplanted mouse model of lung cancer

As shown in Figs. 4 and 5, eNK cells showed a clear antitumor effect when injected intratumorally into subcutaneously transplanted tumors. In contrast, systemic administration of eNK cells resulted in substantial accumulation in the lungs. Notably, this pulmonary accumulation resembled the localized concentration observed following intratumoral injection. Based on this observation and to evaluate their antitumor efficacy, we systematically administered eNK cells to a lung orthotopic transplantation model.

**Fig. 7:**
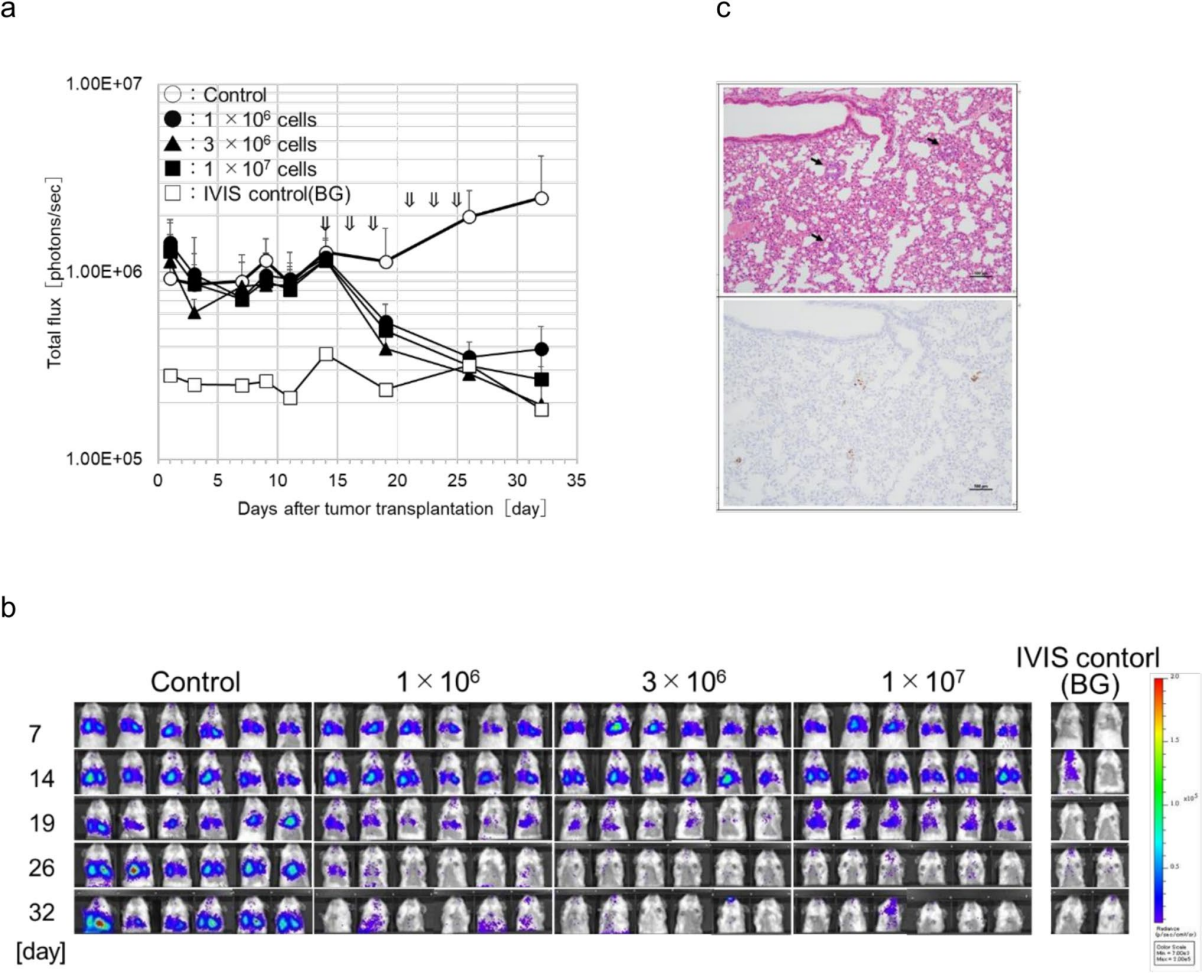
Therapeutic effects of eNK cells in H1975-Luc lung tumor orthotopic graft-bearing mice. **a**, **b** NOG mice were intravenously transplanted with 3 × 10^5^ H1975-Luc tumor cells (day 0). eNK cells at indicated dose were intravenously administered three times a week for 2 weeks from day 14. Tumor growth was measured by IVIS luminescence intensity. Arrows depict the day of eNK cells administration. Data are shown as mean ± SD. For all groups, *n* = 6 except the IVIS control (background) (*n* = 2). **c** At the start of administration 2 weeks after H1975-Luc transplantation, it was confirmed that lung cancer cells had taken root in the lungs (arrows), and therapeutic administration was started. Photographs show H&E stained cells (upper) and Ki67 immunostained cells (lower) in a serial section. Scale bar indicates 100 µm. NOG, NOD/Shi-scid IL-2R gamma (null)

NOG mice were intravenously injected with the H1975-Luc human lung cancer cell line. eNK cells were then intravenously injected at doses of 1 × 10^6^, 3 × 10^6^, and 1 × 10^7^ cells per mouse, three times per week from day 14 to day 25. Seven days after the final administration, eNK cells significantly inhibited tumor growth. At doses of 1 × 10^7^ and 3 × 10^6^ cells /mouse, bioluminescence signals returned to baseline and matched those observed in the IVIS control group without tumor transplantation, suggesting near-complete tumor regression (Fig. 7a). Tumor regression was clearly visualized by IVIS imaging on day 32, 7 days after the last eNK cell administration (Fig. 7b). Histological analysis on day 14 confirmed that H1975-Luc tumor cells were successfully engrafted and distributed within the lung tissue (Fig. 7c). These results strongly suggest that eNK cells possess robust therapeutic efficacy, even when used as monotherapy.

### Enhancement of the antitumor effect of eNK cells by necitumumab-mediated ADCC in A549-Luc lung tumor orthotopic graft-bearing mice

We evaluated whether the antitumor effect of eNK cells could be enhanced via ADCC activity in tumor-bearing model mice with wild-type EGFR-positive A549-Luc cells. eNK cells exhibited a clear dose-dependent tumor growth inhibition in A549-Luc tumor-bearing mice, similar to the effect observed in H1975-Luc tumor-bearing mice. NOG mice were intravenously injected with A549-Luc tumor cells on day 0. eNK cells were administered intravenously at a dose of 5 × 10^6^ cells/mouse, three times per week for 2 weeks starting on day 4. This dose was selected as a partially effective dose based on prior evaluations. Nmab was administered intraperitoneally at a dose of 0.08 mg/body, twice weekly for 2 weeks starting on day 4.

**Fig. 8:**
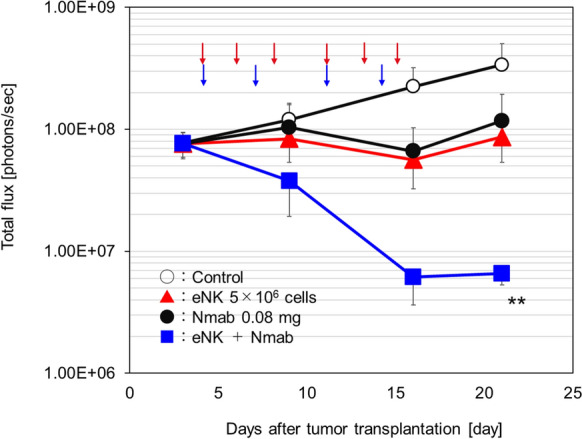
Enhancement of the effects of necitumumab-mediated ADCC by eNK cells in A549-Luc lung tumor orthotopic graft-bearing mice. NOG mice were intravenously injected with 2 × 10^5^ A549-Luc tumor cells (day 0). eNK cells at a dose of 5 × 10^6^ cells/mouse were intravenously administered three times a week for 2 weeks from day 4. Nmab at a dose of 0.08 mg/body was intraperitoneally administered twice a week for 2 weeks from day 4. Arrows depict the day of eNK cells (red) and Nmab (blue) administration. A549-Luc tumor growth was measured by IVIS luminescence intensity. Data are shown as mean ± SD. For all groups, *n* = 5. **; *p* < 0.01 vs control (vehicle) (Dunnett multiple comparison test). ADCC, antibody-dependent cellular cytotoxicity; NOG, NOD/Shi-scid IL-2R gamma (null); Nmab, necitumumab

Both eNK cells and Nmab inhibited tumor growth, and their combination resulted in more pronounced antitumor effect than either treatment alone. These results suggest that the potent antitumor effects of the combination of eNK cells and Nmab are likely mediated by ADCC (Fig. 8).

## Discussion

Immune checkpoint inhibitors (ICIs) such as PD-1, PD-L1 antibodies have shown therapeutic efficacy, including for treating non-small cell lung cancer (NSCLC) [18–20]. However, only about 20% of lung cancers respond to ICIs, highlighting the need for alternative immunotherapies [19]. Moreover, ICIs rely on the presence of tumor-reactive T cells, and many cancer patients experience T cell exhaustion [21, 22]. CAR-T cell therapy has demonstrated therapeutic potential for hematological malignancies. Nevertheless, its efficacy in heterogeneous solid tumors remains limited due to the lack of persistence and exhaustion of CAR-T cells, as well as their limited ability to home in on target tumor sites.

To address these challenges, we have developed eNK cell therapy by generating gene-engineered iPSC-derived NK cells [11] eNK cells offer distinct advantages as a novel immunotherapy for solid tumors, including non-HLA-restricted cytotoxicity, enhanced tumor homing via CCL19 and CCR2B, and robust ADCC activity mediated through high-affinity CD16. Furthermore, as they are derived from iPSCs, eNK cells can be manufactured on a large scale, stored for extended periods, and readily available for immediate use without the need for patient-specific cell collection or processing. These features collectively make eNK cells a promising candidate for allogeneic “off-the-shelf” immunotherapy.

The present study demonstrated the antitumor efficacy of eNK cells in the CDX model and in the PDX model. In the CDX model, eNK cells achieved near-complete tumor regression, whereas in the PDX model, their efficacy as monotherapy was modest, with a 28% tumor volume reduction observed. This discrepancy may reflect the increased heterogeneity and immune-excluded phenotypes of patient-derived tumors, which more closely mimic the complexity of human disease. In the PDX model, eNK cells were administered intratumorally; therefore, this system was not designed to evaluate chemokine‑mediated homing from the circulation or de novo infiltration of eNK cells into tumors. Rather, our histological analyses primarily reflect the local persistence and distribution of administered human cells within the tumor mass. Importantly, immunohistochemistry of treated PDX tumors collected on Day 35 (16 days after the last administration) confirmed the presence of clustered human CD45-positive cells within the tumor parenchyma in eNK-treated mice, while such cells were almost absent in vehicle- and cetuximab-only tumors (Fig. 5e). Notably, the intratumoral distribution pattern of human CD45-positive cells was comparable between eNK monotherapy and the combination group, supporting the interpretation that the enhanced antitumor efficacy observed with cetuximab is consistent with functional augmentation via ADCC. Collectively, these results support the clinical utility of combining eNK cells with therapeutic antibodies (cetuximab or necitumumab) to enhance antitumor activity via ADCC (Figs. 5 and 8). Future studies will be needed to dissect chemokine-mediated homing and infiltration of eNK cells using models and evaluation systems specifically optimized for that purpose.

Although lung cancer models were the primary focus of this study, we have also conducted experiments to evaluate eNK cell infiltration and functionality in a subcutaneous mesothelioma (MPM) model. Preliminary results suggest greater infiltration of eNK cells (human CD45-positive cells) into tumor tissue compared to iNK cells, as well as a tendency for eNK cells to suppress tumor growth under the same conditions where iNK cells showed no notable effect. These findings provide initial evidence of the enhanced functionality of eNK cells, likely mediated by the introduced genetic modifications. The detailed data and results are currently being analyzed and will be presented in a separate manuscript.

eNK cells demonstrated strong cytotoxic activity against various lung cancer cell lines (Fig. 2) and exhibited significant antitumor effects in an in vivo orthotopic lung cancer model. Based on the in vivo distribution characteristics of cell therapy products, it is expected that systemically administered eNK cells will accumulate in the lungs [23–25]. Furthermore, after intravenous administration, eNK cells were primarily localized in the lungs, which likely contributed to their antitumor effects in the orthotopic lung cancer model. This observation supports the feasibility of intravenous administration as a clinical delivery route for eNK cells. The present results suggest that eNK cells have similar properties. In addition, it was found that the number of eNK cells decreased after reaching a peak at 24 h following intravenous administration. This reduced level was maintained for approximately one week. Subsequently the number of eNK cells increased and eventually returned to the level observed at 24 h post-administration, where it remained for up to 28 days. The persistence of eNK concentrations is considered to contribute to adequate efficacy. The mechanism underlying the increase in the number of eNK cells is currently unclear, but it is considered a subject for future investigation. Consistent with these pharmacokinetic findings in normal NOG mice, human CD45-positive cell clusters were still detectable within treated PDX tumors 16 days after the last eNK administration (Fig. 5e), suggesting that administered human cells can persist locally within the tumor microenvironment in tumor-bearing mice.

Additionally, local intratumoral administration of eNK cells in subcutaneously transplanted PDX tumors can be used to model and predict the therapeutic effects of systemic administration. Therefore, the antitumor effect demonstrated by eNK cells in PDX models, which has high predictive value clinically [26, 27], further suggests their potential usefulness in clinical applications.

Long-term safety is also a critical consideration for the clinical translation of eNK cells. In a safety study, the maximum feasible dose of eNK cells was administered intravenously to NOG mice, followed by observation for 39 weeks. Histopathological analysis of major organs, including the heart, liver, spleen, lungs, and kidneys, revealed no tumorigenic or toxicologically significant findings, supporting the safety of eNK cells. These results are included in the Supplementary Materials (Table S1).

In conclusion, eNK cell therapy is a novel and promising treatment option for refractory lung cancer. However, the antitumor effect of eNK cells alone is limited in preclinical models, so further evaluation is necessary to optimize therapeutic outcomes. Combination strategies with other antibody-based drugs or refined dosing schedules may be essential to maximize efficacy and ensure the translational potential of this approach.

## Supplementary Information

Below is the link to the electronic supplementary material.

Supplementary file1 (DOCX 501 KB)

## Data Availability

No datasets were generated or analysed during the current study.
