# Supplementary material for: An innovative treatment for lung cancer using gene-engineered human-induced pluripotent stem cell-derived natural killer cells
Source: Cancer Immunol Immunother. 2026 Mar 31;75(4):129. doi: 10.1007/s00262-026-04370-7 (PMC13038767; doi:10.1007/s00262-026-04370-7)
Supplement: Supplementary file 1 — Supplementary file1 (DOCX 501 KB) [file 262_2026_4370_MOESM1_ESM.docx]

Supplementary Table

Table S1. **Histological Findings in the Tumorigenicity Study of eNK cells**

| **Items** | **Groups** | | | | | |
| --- | --- | --- | --- | --- | --- | --- |
| **Dose (cells in 150 µL HBSS/animal)** | **0** | **2 × 10^7^** | | | | |
| **Observation period (weeks)** | **26** | **4** | **8** | **12** | **26** | **39** |
| **Number of animals tested　(male/female)** | 5/5 | 5/5 | 5/5 | 5/5 | 5/5 | 4/4 |
| **Gross pathology (male/female)** | 0/0 | 0/0 | 0/0 | 0/0 | 0/0 | 0/0 |
| **Histopathology　(male/female)** | 0/0 | 0/0 | 0/0 | 0/0 | 0/0 | 0/0 |

1. Numbers in the gross pathology and histopathology indicate the number of animals with findings.
2. Organs examined in histopathology: Lungs/bronchi; liver; heart; kidneys; testes; ovaries, brain; sternum; sternal bone marrow; femur; femoral bone marrow; submandibular lymph nodes; mesenteric lymph nodes; spleen; injection site
3. HBSS, Hank’s balanced salt solution.

Table S2. **Blinded qualitative scoring of intratumoral human CD45-positive cell clusters in PDX tumors.**

| Group | n | Cluster present | Cluster absent |
| --- | --- | --- | --- |
| Vehicle | 3 | 0 | 3 |
| eNK | 4 | 4 | 0 |
| Cmab | 5 | 0 | 5 |
| eNK+Cmab | 4 | 4 | 0 |

Whole-slide images of human CD45 immunohistochemistry were evaluated in a blinded manner by two independent evaluators. The presence of clustered human CD45-positive cells within viable tumor parenchyma was recorded as a binary outcome (present/absent) for each tumor. “Present” was defined as at least one intratumoral cluster composed of multiple CD45-positive cells, whereas isolated single cells or background staining alone were scored as “absent”. The two evaluators’ assessments were fully concordant.

Supplementary Figure

Fig S1. **Histological evaluation of PDX tumors**

a
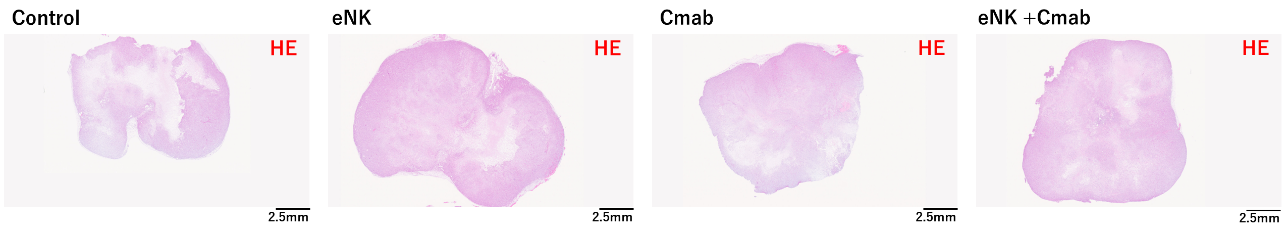


b


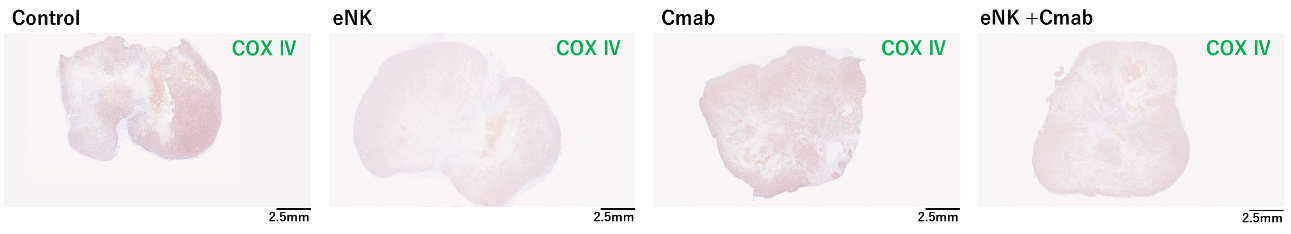


Representative H&E staining (a) and human COX IV immunohistochemistry (b) in PDX tumors harvested on Day 35 (16 days after the last administration) from vehicle, eNK, cetuximab, and combination groups. Whole-slide images were acquired using a Hamamatsu NanoZoomer digital slide scanner.
